# Criminal law regulation of cyber fraud crimes—from the perspective of citizens’ personal information protection in the era of edge computing

**DOI:** 10.1186/s13677-023-00437-3

**Published:** 2023-04-24

**Authors:** Yu Zhang, Haoyun Dong

**Affiliations:** 1 Criminal Justice School, Zhongnan University of Economics and Law, Wuhan, 430073 China; 2 School of Criminology, People’s Public Security University of China, Beijing, 100038 China

**Keywords:** Internet fraud crime, Cloud computing, Analytic hierarchy process, Fuzzy comprehensive evaluation, Personal information security, Governance capacity

## Abstract

Currently, cloud computing provides users all over the globe with Information and Communication Technology facilities that are utility-oriented. This technology is trying to drive the development of data center design by designing and building them as networks of cloud machines, enabling users to access and run the application from any part of the globe. Cloud computing provides considerable benefits to organizations by providing rapid and adaptable ICT software and hardware systems, allowing them to concentrate on creating innovative business values for the facilities they provide. The right to privacy of big data has acquired new definitions with the continued advancement of cloud computing, and the techniques available to protect citizens’ personal information under administrative law have managed to grow in a multitude. Because of the foregoing, internet fraud is a new type of crime that has emerged over time and is based on network technology. This paper analyzed and studied China’s internet fraud governance capabilities, and made a comprehensive evaluation of them using cloud computing technology and the Analytic Hierarchy Process (AHP). This paper discussed personal information security and the improvement of criminal responsibility from the perspective of citizens’ information security and designed and analyzed cases. In addition, this paper also analyzed and studied the ability of network fraud governance in the era of cloud computing. It also carried out a comprehensive evaluation and used the fuzzy comprehensive evaluation method to carry out the evaluation. A questionnaire survey was used to survey 100 residents in district X of city Z and district Y of the suburban area. Among the 100 people, almost all of them received scam calls or text messages, accounting for 99%, of which 8 were scammed. Among the people, more than 59.00% of the people expressed dissatisfaction with the government’s Internet fraud satisfaction survey. Therefore, in the process of combating Internet fraud, the government still needs to step up its efforts.

## Introduction

A novel method of providing information communications technology to businesses is called cloud computing [1]. It is based on the premise that businesses do not need to invest in hardware, software, or network infrastructure to run mission-critical applications. Without spending money on new facilities, hiring new staff, or licensing the latest software, organizations can expand their application of information and communication technology capacity or add new functionality by using cloud-based facilities. Cloud computing has become increasingly popular in recent years. There are many different types of cloud computing, including online file storage services and servers that are simple to customize. Then, when consumers have access to these electronic tools, they can use them, however, suits their needs, such as creating virtual servers for web hosting or software development or creating online internet-based backups. Most of these uses are generally benign, but the usage of technology does carry an essential hazard, as total security remains a top priority for providers of cloud-based services and those who use cloud storage.

Due to the emergence of cloud computing the Internet has certainly greatly facilitated people’s daily life. However, a large number of fraudulent behaviors have appeared on the Internet, which has caused social harm that cannot be underestimated. China’s current legislation does not have clear provisions on the crime of cyber fraud, which is only limited to the crime of fraud. Therefore, judicially, the network fraud crime is identified and punished as a fraud crime. However, because the complexity and harm of Internet fraud are much greater than that of fraud, there are other aspects to consider when committing Internet fraud. In recent years, cyber fraud crime is characterized by virtualization of space, concealment of behavior, low age, low culture, regionalization, industrialization of the cyber fraud crime chain, diversification of fraud practices, and rapid renewal. Network fraud not only causes economic losses to the people but also seriously affects the people’s sense of happiness and gain.

Cyber fraud crimes occur year by year, causing huge losses to people. In almost all telecom fraud crimes, the criminals use bank cards and mobile phone cards opened by others’ ID cards. It is difficult for public security organs to seize the true information of the suspects through call records, fund transfers, etc. Telecom fraud crimes not only infringe on the property interests of the people but also erode information security and financial security, resulting in related upstream and downstream crimes such as infringement of citizens’ personal information, helping information network crime, etc. Because of the increasing number of cyber fraud cases in China in recent years, in 2021, courts across the country will conclude more than 25,000 such cases in the first instance, and more than 61,000 defendants will be sentenced to punishment. Chinese legislation has not yet established it separately, so traditional methods are used in practice. Therefore, this paper discusses the standard of determining criminal responsibility, hoping to be helpful to the problems existing in China’s judicial practice.

Although these days, the popularity of the Internet and cloud computing technology have provided convenience for people’s lives, the Internet has also provided a convenient platform for criminals to commit crimes. Internet fraud is one of the most common property crimes. To accurately detect and prevent cyber fraud, Zhang et al. used the Apriori algorithm to mine the association rules of the information in some samples of cyber fraud cases. Its attribute fields for searching association rules were crime location, crime scope, crime time, number of cases, and degree of loss [2]. Since it was influenced by research on the third-person effect of social media and online media, Wei et al. examined the negative impact of increased mobile internet fraud on users’ social relationships and their possible responses to increasing fraud [3]. Chang et al. aimed to evaluate novel coronavirus pneumonia (COVID-19)-related fraud cases to identify cognitive heuristics that influence decision-making under the stress of crisis conditions. The findings of this study can help individuals avoid fraud victimization by understanding psychological vulnerabilities they may not be aware of in crisis conditions [4]. Dzomira et al. analyzed the online banking fraud vigilance of South African banking institutions to the general public. It centers on the theory of routine activity, which is a theory of criminology. Qualitative content analysis was used as a research technique to interpret the textual data of each bank’s website through a systematic classification process of encoding and identifying themes or patterns to gain insight into the banking industry’s online banking fraud vigilance [5]. Starostenko et al. specifically discussed issues related to the nature and methods of fraud using information and telecommunication technologies. Official statistics of global Internet crimes in 2019 were considered and analyzed, as well as the material damage caused by these crimes [6]. However, their research has not yet explored methods for assessing Internet fraud governance capabilities.

Besides cloud computing, the AHP allows decision-makers to model problems as hierarchies containing relationships between goals and alternatives. Therefore, many scholars have focused on controlling and evaluating cyber fraud crimes. Felipe et al. attempted to fill this void by proposing a supplier-based segmentation model capable of aggregating qualitative and quantitative criteria. The suggested model can be viewed as a decision-support system that aggregates expert qualitative judgments and quantitative historical performance metrics and offers recommendations for improving supplier-buying firm relationships [7]. Sedighi et al. identified the critical success factors (CSFs) for implementing knowledge management in the Iranian energy sector. By using the AHP method and based on an analysis with the designers of the Iranian energy sector, the relative quantitative weights for implementing knowledge management in 8 major CSFs were determined [8]. Jagtap et al. pioneered the use of AHP in the identification of critical equipment in thermal power plants. The criticality analysis for this AHP-based analysis took four criteria into account: the impact of equipment breakdown on power generation, the environment and safety failure regularity, and maintenance costs [9].

In addition, Ahmed et al. proposed an AHP method based on goals. It delegated pairwise comparison values using field data collected from Mumbai’s road network, which included 28 road segments [10]. Hurley J S studied the evaluation of complex network security issues, and the key to evaluation was to determine factors and weights. To improve the accuracy, he proposed an AHP-based evaluation model to evaluate the network [11]. However, the use of APH needs to take into account many aspects and consider the influencing factors of the display. Yusoff et al. used the analytic hierarchy process to evaluate large-scale open online courses [12]. Santis et al. studied the case of Brazilian railway operators with an experimental fuzzy analytic hierarchy process [13]. Based on the above, this paper first describes the contents related to personal information security and then describes edge computing. At the same time, it also introduces the evaluation system of Internet fraud governance ability in detail. Additionally, it discusses the improvement of criminal responsibility from the perspective of citizens’ information security by designing and analyzing cases. This paper performs several experiments by studying the ability of network fraud governance in the era of cloud computing. Through the questionnaire analysis of 100 citizens, this paper explores the governance ability of Internet fraud in the era of edge computing. The contributions of this research work are listed as under:

- This paper discusses the governance of network fraud in Z city by case analysis and fuzzy comprehensive evaluation in the era of cloud computing.
- The innovation of this paper is to combine cloud-based AHP with the evaluation method of governance capability and conduct a more detailed analysis of the evaluation system of Internet fraud governance capability and the comprehensive evaluation of Internet fraud governance capability.

The remaining part of the paper is structured in the following manner: The evaluation method of internet fraud governance capability using edge computing technology is deliberated in Sect. "Evaluation of internet fraud governance capability using edge computing". Detailed discussion of the role of cloud and edge computing for information fraud and security is also given in this section. Moreover, two algorithms are suggested that will enhance the internet security for information. Experimental results and discussion are given in Sect. "Experiments on internet fraud governance capability in the era of cloud-edge". Finally, Sect. "Conclusions" summarizes the major outcomes of this study.

## Evaluation of internet fraud governance capability using edge computing

### Personal information security

**Fig. 1:**
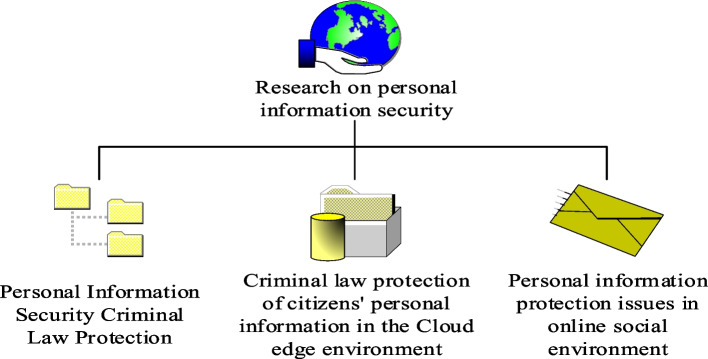
Research trends on personal information security

As far as the current research situation is concerned, the research on personal information security generally includes several major trends in Fig. 1. They are the protection of personal information security by criminal law, the protection of citizens’ personal information under the network environment by criminal law, and the protection of personal information under the network social environment.

The first is the protection of personal information security by criminal law. In legislation, criminal law has stipulated the comprehensive protection of citizens’ personal information. However, in judicial practice, there are many differences in how to implement it. At present, the definition of personal information and the definition of the subjective guilt of personal information crimes are controversial in academic circles, and further research and clarification are needed. The second is the criminal law that protects citizens’ data. The spread of information does not need to be far away, and the spread can be accepted, so the spread and communication are faster and more convenient. Therefore, the illegal and criminal activities of personal data on the Internet are becoming more and more intense, and the geographical scope is not large, which often affects many countries or regions. To effectively deal with the increasing data and information leakage problem, the security of the network has been enhanced. According to the basic principle of the ultimate means of criminal law, the security of citizens’ information can be protected by measures such as the improvement of relevant regulations, the expansion of information crime subjects, and the standardization of the objectivity of information crimes [14, 15]. The third is to discuss the protection of personal information in the network society. At present, academic research mostly discusses how to effectively protect the security of personal information in social networking from the perspectives of sociology, management, and law, but few people discuss it from the perspective of criminal law [16].

From the current situation of China’s criminal legislation, the promulgation of the Criminal Law Amendment (XI) has further strengthened the protection of citizens’ information security under China’s existing criminal law. However, the relevant judicial interpretation has not yet been formulated, which has led to different opinions among judges in different regions during the trial process, thus affecting the legal authority of China. The criminal protection of personal data in Chinese criminal law academic circles has always been divided, but due to its shortcomings, there are many defects in China’s current criminal legislation system, which gives criminals an opportunity. In China, most Internet users have experienced the problem of personal information being violated, which not only affects their daily life but also produces new violations due to the advancement of technology [17]. In the network society, how to ensure the security of personal information to prevent others from illegal acquisition, malicious intrusion, improper collection, and even destruction are the problems that need to be solved urgently at present.

### Edge Computing for Internet Fraud Governance Capability

Edge computing mainly refers to providing computing services nearby at the side close to the object or data source to generate faster network service response and meet the real-time application and data protection requirements. Recently, the concept of edge computing is extremely hot, and some people even think that edge computing will be the “terminator” of cloud computing.

Taking the IoT scenario as an example, devices in the IoT generate a large amount of data in the process of uploading to the cloud for processing; it will cause huge pressure on the cloud. In order to share the pressure of the central cloud node, edge computing nodes can be responsible for their own data calculation and storage.

However, because most of the data is not one-time data, those processed data still need to be gathered from the edge nodes to the central cloud. The central cloud has undergone big data analysis and mining, data sharing, and algorithm model training and upgrading. The upgraded algorithm is pushed to the front end for updating and upgrading, so as to complete the closed loop of autonomous learning. At the same time, the data at the storage edge needs to be backed up. When an accident occurs in the edge computing process, the data stored in the cloud will not be lost.

In other cases, cloud computing and edge computing work together to form a complementary and cooperative relationship. To better meet the needs of various application scenarios, edge computing must collaborate closely with cloud computing. Edge computing is primarily responsible for processing some real-time and short-term data, as well as real-time processing and execution of local businesses, to provide high-value data to the cloud; cloud computing is willing to take responsibility for computing tasks that edge nodes are not capable of performing. At the same time, it is in charge of processing non-real-time, long-period data, optimizing the output business rules or designs, and pushing them to the edge. Therefore, edge computing can more meet local needs and complete the full life cycle management of applications.

The open platform of the Internet of Things is divided into four levels: data collection, data storage, and data service and data management. Among them, information collection involves the sensing layer and network level of the Internet of Things system, which requires the Internet of Things open platform to adopt a unified access mode when facing a large number of sensing terminals and complex network environment; Data storage and data service system must have functions similar to middleware, which can combine complex logic and data processing, and provide unified services for data analysis and computing services.

This architecture model can make full use of the resource integration, management, scheduling and other characteristics of cloud computing to provide high-performance and scalable distributed communication, storage and computing capabilities for distributed communication, storage and computing, and combine it with service-oriented concepts to provide unified support for the overall data of the system. Through life cycle management, interaction management, reliability and availability management, loose architecture is implemented in the whole system.

**Fig. 2:**
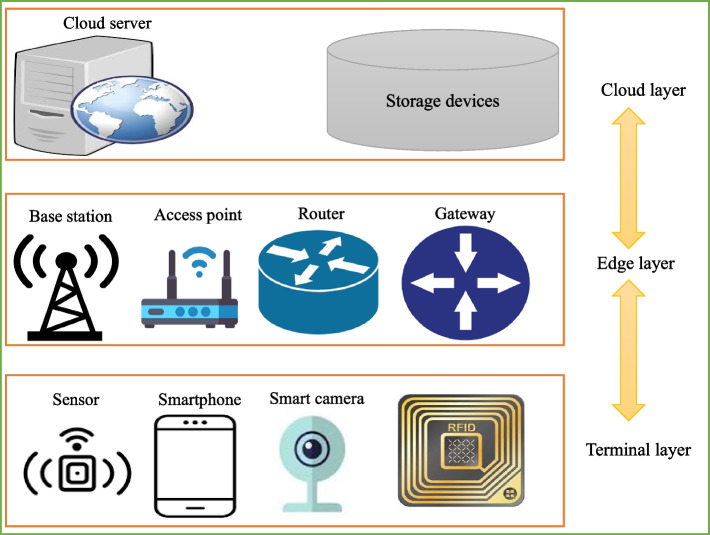
Structure of cloud-edge collaboration for internet fraud governance capability

The structure of the proposed cloud-edge collaboration for internet fraud governance capability is shown in Fig. 2 and generally is divided into three layers including terminal, edge and cloud computing.

The following is a brief introduction to the composition and functions of each layer in the edge computing architecture.

#### Terminal layer of edge computing for internet fraud governance capability

The terminal layer includes those devices that are attached to the edge network, like mobile terminals (MT) and several IoT or sensor-based devices including sensing devices, smart cars, smartphones, smart cameras, smart homes etc. The device is both a data user and a data source at the terminal layer [26, 27]. To decrease terminal service delays, only the perspective of the numerous terminal devices is taken into account, rather than computer resources. As a consequence, hundreds of billions of terminal layer devices gather various types of raw data and send it to the top layer, where it is saved and determined.

#### Boundary/edge layer of edge computing for internet fraud governance capability

This layer is the heart of the three-tier design, which is placed at the network’s edge and is made up of edge nodes that are broadly distributed among devices connected and clouds. Most frequently, it consists of base stations, edge routers, access points, switches, gateways, etc. The edge layer facilitates terminal device downward access and retail chains and computes information uploaded by terminals. Join the cloud and send the processed information there [18]. Because this layer is closer to the user, data transfer to the edge layer is better suited for real-time data assessment and digital signal processing, which makes it more effective and safer than cloud computing.

#### Cloud layer of edge computing for internet fraud governance capability

Cloud computing remains the strongest data processing facility among the federated facilities of cloud-edge computing. The cloud computing layer is made up of many high-performance servers and storage devices that have strong computing and storage functionality. It can be useful in areas that require large amounts of information assessment, such as regular maintenance and corporate decision assistance. The cloud computing center can completely store the edge computing layer’s provided results, as well as complete analysis tasks that the edge of the network layer cannot control and data processing that incorporates global information. Furthermore, the cloud module can dynamically adjust the edge computing layer’s deployments and algorithm by the control policy [29, 30]. The notations used in this paper can be highlighted in Table 1.

**Table 1:** Notations used in this paper

S No	Notation used	Description of notation
1	*ID*	Identity of circuit
2	*ExitNode*	The node used for exit the traffic
3	*Q*_*ok*_	Specific factor at the second level, where o is the start point and k = 1,2,3…
4	*Q*_*o*_	Initial factor
5	*Q*_*k*_	Final factor
6	*µ*_*max*_	Maximum eigenvalue
7	*m*	Square root
8	*E*	Priority vector
9	*S*	Decision matrix
10	*VO*	Consistency index
11	*VT*	Consistency ratio
12	*TO*	Random consistency index

For the peak path selection of internet fraud, we designed algorithm 1, which selects routers in a specific order given a desired path length of 3 by default. Tor does not use the same router for the same path twice. According to this Algorithm, Tor selects an exit node, an entry node, and then a middle node in that order to create a circuit.
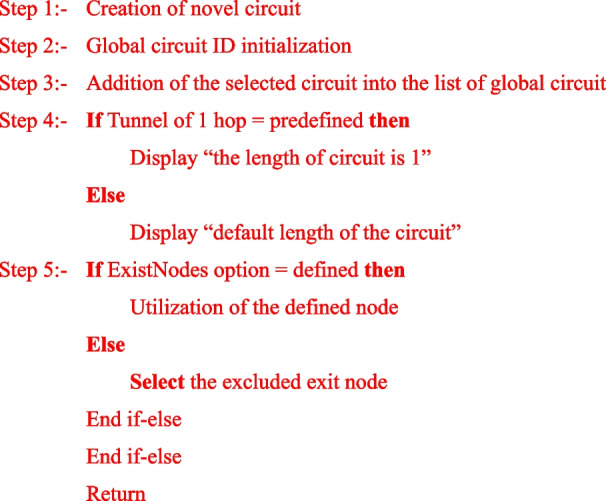



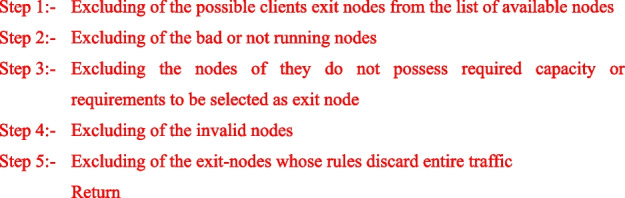


### Evaluation system of internet fraud governance using edge-cloud computing

**Fig. 3:**
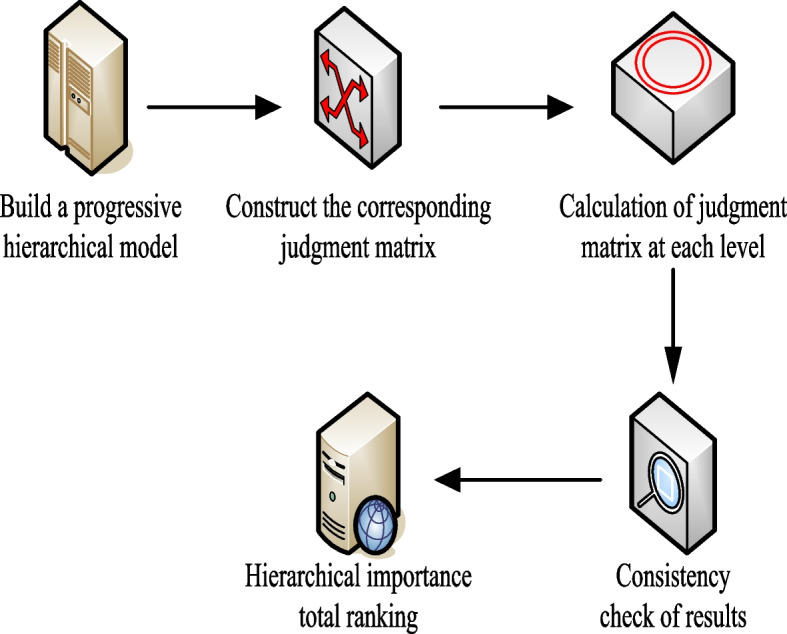
Operation steps

AHP is a kind of evaluation problem that is suitable for evaluating objects with multiple attributes, complex structure, and inability to fully use quantification. Through the evaluation of China’s fraud governance capacity, an evaluation index based on this index system is established. The influence degree of each factor is analyzed, which provides a basis for the determination of the current level of China’s Internet fraud governance [19]. The AHP method is used to assign weights to each indicator. The specific operation process is shown in Fig. 3.

#### Establishment of a progressive hierarchical model

The research goal is to divide the indicators to measure the ability of network fraud governance into the target layer, the criterion layer, and the indicator layer. Among them, early warning and prevention ability, case handling ability, and governance effect are the three first-level indicators. The second aspect is publicity and education, early warning and handling, supervision and management, system adjustment and improvement, emergency response capabilities, case investigation capabilities, prevention effects, and public satisfaction. In addition, according to each secondary indicator, 25 specific sub-projects have been established [20, 28].

#### Construction of the corresponding judgment matrix

According to the detailed process of AHP, each factor of the same layer is compared and then scored with the corresponding ratio scale. In addition, according to the relative importance, a comparative conclusion is drawn, which represents the importance of each element of the next stage in the next stage. For example, it is assumed that the index at the first level is *S*, while the specific factor at the second level is $Q_{ok} (o,k = 1,2,3,4,5......)$, then each specific factor *Q* at the second level can be compared pair-wise with the relative importance of the first level. This leads to *S-Q*, as shown in Table 2.

**Table 2:** 1–9 Ratio scale

**Scale 1**	The specific meaning of factor $Q_{o}$ than factor $Q_{k}$
1	Both elements are equally important
3	Factor $Q_{o}$ is slightly more important than factor $Q_{k}$
5	Factor $Q_{o}$ is stronger and more important than factor $Q_{k}$
7	Factor $Q_{o}$ is strongly more important than factor $Q_{k}$
9	Factor $Q_{o}$ is extremely important than factor $Q_{k}$
2,4,6,8	Represents the median value of two adjacent judgments

$q_{ok}$ is the ratio between $Q_{o}$ and $Q_{k}$, representing the relative importance of these two elements for indicator Q sex ratio. Usually $Q_{ko} = 1/q_{ok}$

#### Calculation of judgment matrix at each level

The third step is to use a specific method to find the maximum eigenvalue $\mu_{\max }$ of each level and a specific weight (feature vector priority) *E*.

First, the elements in the pre-constructed judgment matrix are multiplied horizontally.

1 $$Q_{o} = Q_{o1} * Q_{o2} * Q_{o3} * ...... * Q_{ok}$$

The calculation result of the previous step is rooted, and then the method of rooting is used to express $Y_{o}$. Among them, *m* in the square root is the sum of all factors.

2 $$Y_{o} = \sqrt[m]{{Q_{o} }}$$

The relative weights of each factor and the previous stage are obtained. That is, the results of the previous stage are compared with the sum of the previous stage, and the relevant weights between the factors are obtained.

3 $$Eo = \frac{Yo}{{\sum\limits_{o = k}^{m} {Yo} }}$$

The relative weights of $E = [E_{1} ,E_{2} ,E_{3} ,...,E_{o} ]$ for each index factor are given. Similarly, the relative weights of each factor at each level and the previous criterion are obtained, and the relative weights (i.e.: total ranking weights) are also obtained.

#### Consistency test of the results

The consistency test results of the model reflect the coordination of various factors in the indicator system. The specific process is as follows: first, the largest eigenvalue is obtained in Eq. 4.

4 $$SE = \left[ {\begin{array}{*{20}c} {Q11} & {Q12} & {Q13} & {...} & {Q1k} \\ {Q21} & {Q22} & {Q23} & {...} & {Q2k} \\ {Q31} & {Q32} & {Q33} & {...} & {Q3k} \\ {...} & {...} & {...} & {...} & {...} \\ {Qo1} & {Qo2} & {Qo3} & {...} & {Qok} \\ \end{array} } \right] * \left[ {\begin{array}{*{20}c} {Q1} \\ {Q2} \\ {Q3} \\ {...} \\ {Qo} \\ \end{array} } \right]$$

In above equation,* S* is the decision matrix. *E* is a priority vector, that is, weights.

Similarly, the problem $\mu \max = \frac{1}{n}\sum\limits_{o = 1}^{m} {\frac{{S_{o} * E_{o} }}{{E_{o} }}}$ is solved, where *n* represents the order of the judgment matrix.

Based on $VO = \frac{\mu \max - n}{{n - 1}}$, the consistency index is solved, where *m* represents the order of the matrix; *VO* is the consistency index; *VT* is the consistency ratio; *TO* is the random consistency index.

Finally, the agreement ratio is calculated based on $VT = \frac{VO}{{TO}}$, and the resulting calculation is compared to 0.1. If $VT < 0.1$, it indicates that the coordination among the elements in the indicator system is good; if $VT > 0.1$, it indicates that the coordination among the elements in the index system is poor. Among them, $VO$ is the result calculated in the previous step, and $TO$ is the given known value as shown in Table 3.

**Table 3:** Comparison of the values of $TO$

Order n	TO
2	0.00
3	0.59
4	0.90
5	1.13
6	1.25
7	1.33
8	1.42
9	1.46
10	1.50

#### Total ranking of hierarchical importance

A second-level indicator is used as a case, and the calculation process of the relevant weights of the indicators of “publicity and education”, “early warning processing work”, “supervision work”, “system adjustment and improvement” and the “early warning and prevention ability” criterion is described in detail:

First, according to the results of experts’ scoring, the evaluation criteria of “early warning and prevention ability” in the previous stage are established, and they are compared and judged. Construct a decision matrix about the “alert defense capability” in the previous layer.

In the previous stage, the normalized relative importance vector $E^{0} = E_{o}^{0}$ for each feature is found. The square root method is usually used. First, perform a horizontal product on each element in the discriminant matrix, and then perform a square root operation. Next, the above results are orthogonalized by first adding the above results and then comparing their values with the summed value.

The weight of each index obtained from the following:

The largest eigenvalue is calculated and checked for consistency. First, find the product of the weight of each element and the judgment matrix. After that $VO$ is solved again, and $VT$ is checked for consistency:

In conclusion, the calculated value of the sigmoid decision matrix constructed in this paper has been verified by consistency. In the same way, the weights of the first-level indicators and other second-level indicators are calculated.

Similarly, the weights of the three-level indicators are calculated, and the composite weight of each indicator in the network governance capability indicator system is obtained according to the calculated weights of the first-level, second-level, and third-level indicators.

### Comprehensive evaluation of internet fraud governance capabilities

**Fig. 4:**
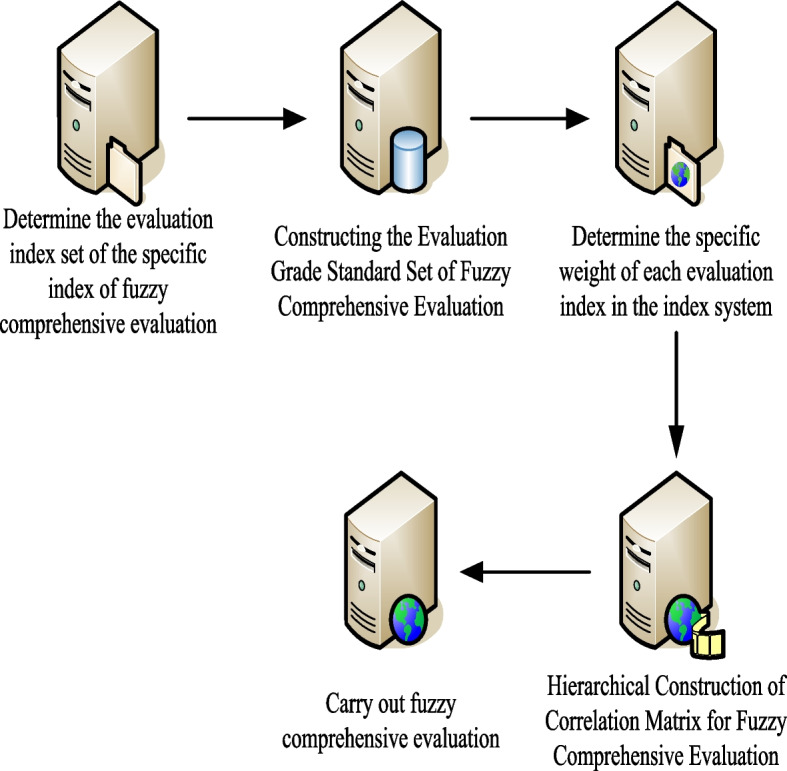
Operation process of fuzzy comprehensive evaluation method

The fuzzy comprehensive scoring method is a comprehensive evaluation method based on fuzzy mathematics. It can transform qualitative evaluation problems into quantitative evaluation problems, that is, quantitative evaluation of unclear targets through fuzzy mathematics of fuzzy mathematics. It can improve the credibility of the evaluation, and make the evaluation process and results clearer and more systematic [21, 22]. By using this method, the ability to govern cyber fraud is assessed and the qualitative content turns into quantitative results. Based on the index system of Internet fraud governance capability, the fuzzy comprehensive evaluation method is used to evaluate China’s Internet fraud governance capability. According to the evaluation results, it conducts corresponding analysis, and thus deeply analyzes the problems and defects in China’s Internet fraud governance. The specific workflow of the fuzzy comprehensive evaluation method is shown in Fig. 4.

**Fig. 5:**
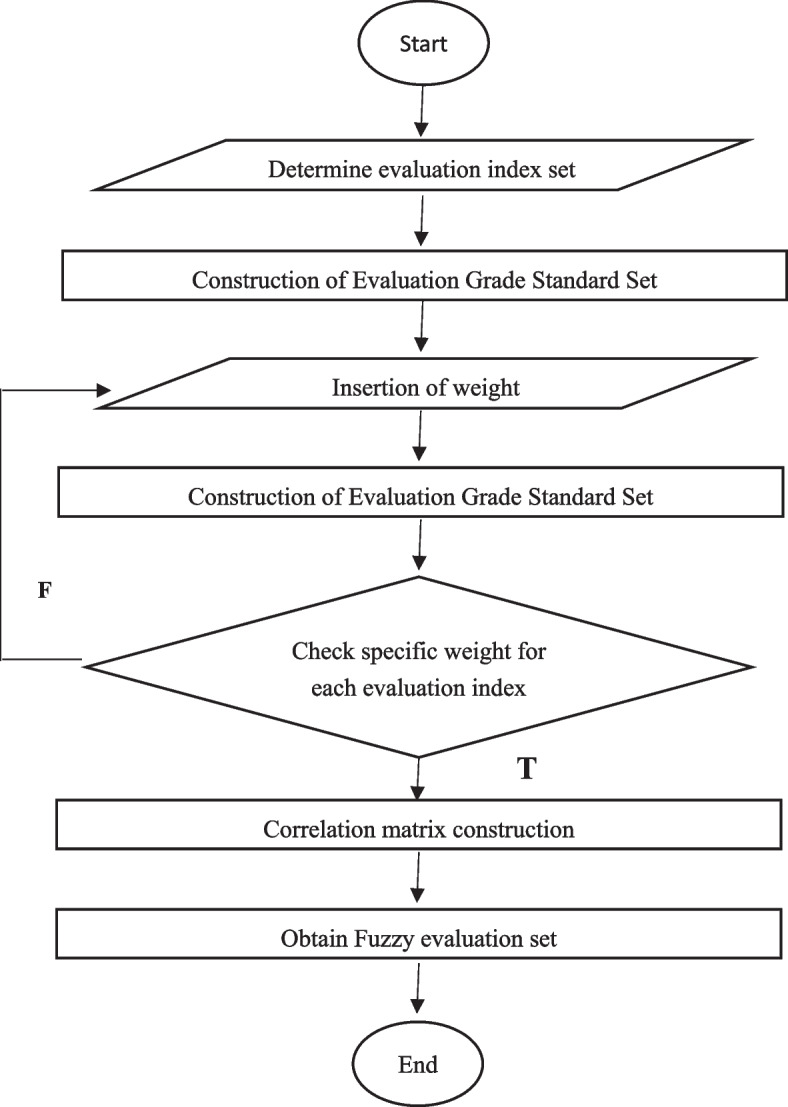
Flowchart of fuzzy comprehensive evaluation

The evaluation indicators in this paper include basic information of citizens (gender, age, monthly income), investigation related to fraud experience, the number and amount of fraud received. In addition, citizens’ satisfaction with the government in combating Internet fraud, and whether the government has taken an active role in combating Internet fraud. The flowchart of the fuzzy comprehensive evaluation can be highlighted in Fig. 5.

Each step of the above flowchart can be explained in this section:

- Determination of evaluation index set of specific index of fuzzy comprehensive evaluation: Evaluation index set $I = \{ i_{1} ,i_{2} ,i_{3} ,...,i_{m} \}$, and *m* represents the number of evaluation indicators at the same level.
- Construction of Evaluation Grade Standard Set for Fuzzy Comprehensive Evaluation: The evaluation level set $B = \{ b_{1} ,b_{2} ,b_{3} ,...,b_{m} \}$. *M* represents the number of evaluation levels, generally 5.
- Determination of the specific weight of each evaluation index in the index system: The evaluation index weight *S* is the weight vector of each level index, usually expressed as $(s_{1} ,s_{2} ,s_{3} ,...,s_{m} )$. The weights satisfy $\sum\limits_{o = 1}^{m} {s_{o} = 1} ,s_{o} \ge 0$ according to the principles of normality and non-negativity.
- Hierarchical construction of correlation matrix for fuzzy comprehensive evaluation: The fuzzy comprehensive evaluation matrix reflects the fuzzy mapping from *I* to *B*. *B* represents the evaluation level, and *I* consists of a single evaluation index set $i_{o} (o = 1,2,3,...,m)$.
  5 $$g:I \to G(B)\begin{array}{*{20}c} {} & {} \\ \end{array} i_{o} \to (t_{o1} ,t_{o2} ,t_{o3} ,...,t_{on} )$$

Therefore, the fuzzy relationship evaluation matrix *T* can be induced by the fuzzy map *f*: $T = (t_{ok} )_{m \times n}$.

6 $$T = \left[ {\begin{array}{*{20}c} {t_{11} } & {t_{12} } & {t_{13} } & {...} & {t_{1k} } \\ {t_{21} } & {t_{22} } & {t_{23} } & {...} & {t_{2k} } \\ {t_{31} } & {t_{32} } & {t_{33} } & {...} & {t_{3k} } \\ {...} & {...} & {...} & {...} & {...} \\ {t_{o1} } & {t_{o2} } & {t_{o3} } & {...} & {t_{ok} } \\ \end{array} } \right]_{m \times n} (o = 1,2,3,...,m;k = 1,2,3,...,n)$$

- Fuzzy comprehensive evaluation: The evaluation weight vector *S* of step (3) and the fuzzy relationship evaluation matrix *T* of step (4) are multiplied, and finally a fuzzy comprehensive evaluation set is obtained.
  7 $$Q = S \bullet T = (s_{1} ,s_{2} ,s_{3} ,...,s_{m} ) \bullet t_{ok} = (q_{1} ,q_{2} ,q_{3} ,...,q_{m} )$$
- Similarly, multi-level fuzzy comprehensive evaluation can be carried out on this basis. First, fuzzy comprehensive evaluation is carried out on the elements of a certain level, and then the hierarchical model is used to gradually increase. Finally, the overall evaluation result is obtained.

## Experiments on internet fraud governance capability in the era of cloud-edge

In the preceding section of the paper, simulations of the aforementioned algorithms and numerical simulations are carried out to validate our proposed approaches using cloud computing. We obtained the following information about the nodes from the Tor network Table 4:

**Table 4:** Experimental parameters

Number of onion routers	1513
Maximum bandwidth in the network at that time was around	5.2 MB/s
Bandwidth of 90% onion routers was	> 350 KB/s
Quality of input data	6 MB
Background white noise	-115dBm

### Influence of Low Internet Fraud Governance Capability

**Fig. 6:**
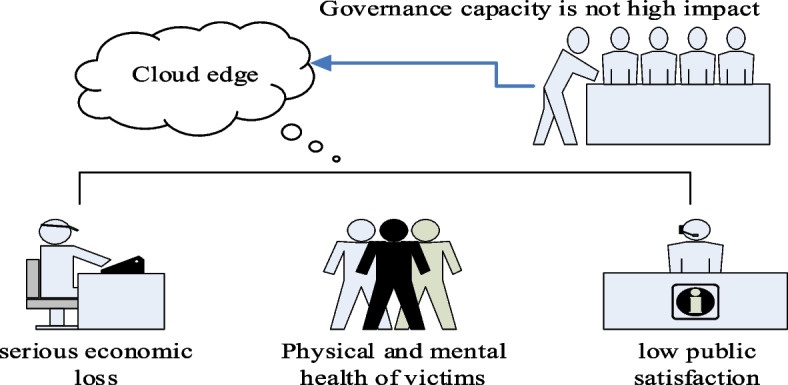
Impact of low governance capacity

In recent years, as the state and various departments have paid more and more attention to the management of cyber fraud, they have carried out in-depth discussions on it. Through a series of rectification and reflection, its management level has been significantly improved. The police have stepped up their investigations into the case, and banks, telecommunications operators, and Internet companies have also stepped up supervision. It is worth mentioning that although the number of crackdowns has increased year by year, the number of crimes remains high. This poses a huge threat to the safety of life and property of the country and the people, and people’s satisfaction is hovering at a low level. New problems continue to emerge without radical cure, which leads to a mismatch between governance capabilities and new situations [23, 24]. It is mainly manifested in several aspects in Fig. 6.

**Fig. 7:**
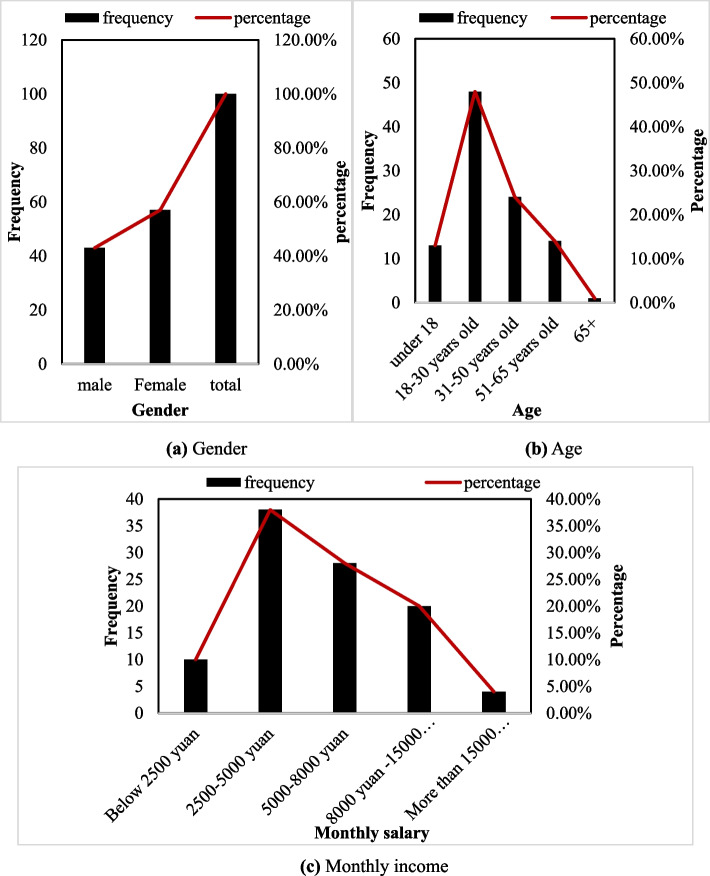
Basic information of the surveyed citizens. a Gender, b Age, c Monthly income

On the basis of the above evaluation indicators, this paper took the ordinary people of City Z as the object, and randomly selected 100 citizens (50 people in each district) in the two areas of District X and District Y of suburban area of City Z. Its basic situation is shown in Fig. 7.

It can be seen from Fig. 7(a) that there were 43 males (43.00%) and 57 females (57.00%) in this survey. From the data in Fig. 7(b), it can be known that the age was generally between 18–30 years old, and there were 48 people (48.00%). It can be seen from Fig. 7(c) that most of the annual incomes were between 2,500–5,000 yuan. There were 38 people (38.00%), and only 4 people (4.00%) had a monthly income of more than 15,000 yuan.

**Fig. 8:**
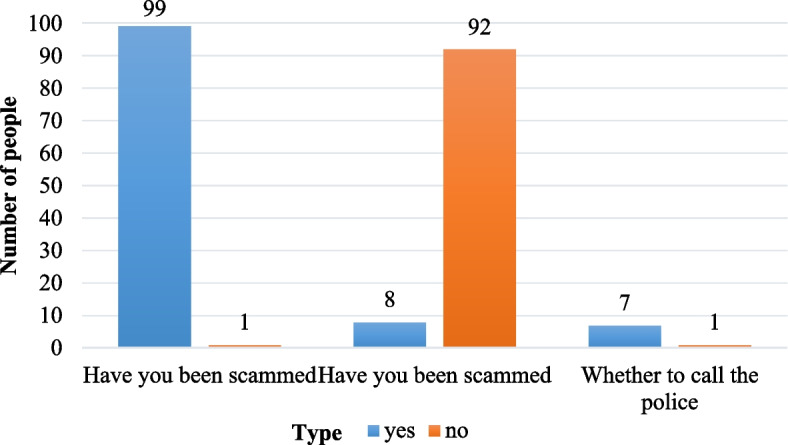
Investigation on Fraud Experience

Figure 8 is related data on some of the scams. It was found that 99 people (99.00%) had received fraudulent phone calls or text messages; 8 people (8.00%) were defrauded, and 7 people (87.5%) chose to report to the police after being defrauded. It can be concluded that the public had a certain awareness of vigilance.

**Fig. 9:**
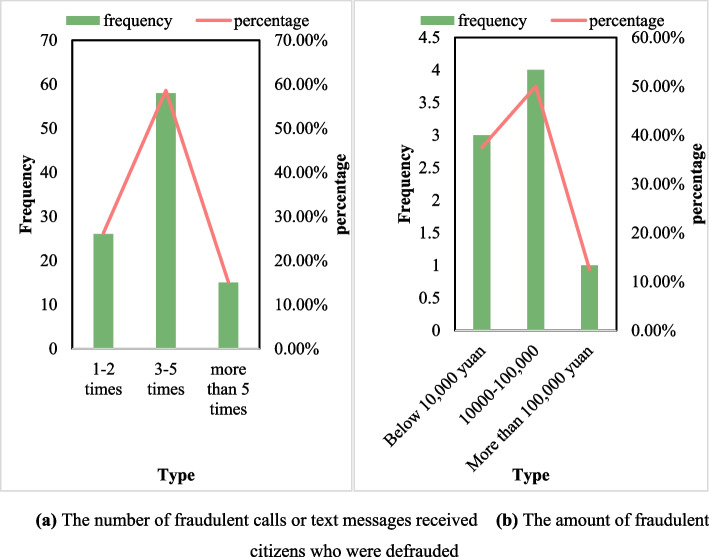
The number of frauds and the amount of fraud. **a** The number of fraudulent calls or text messages received. **b** The amount of fraudulent citizens who were defrauded

Figure 9 is related to the number of times of fraud and the amount of fraud. In Fig. 9(a), a survey was conducted on 99 citizens who had received fraudulent calls or text messages, and it was found that 26 people received 1–2 fraudulent calls or text messages (26.26%); 58 people received 3–5 times (58.59%); 15 people received more than 5 times (15.15%).

In Fig. 9(b), there were 8 people who had been defrauded before; 3 people (37.50%) were deceived with an amount of less than 10,000 yuan; 4 people (50.00%) were defrauded with an amount ranging from 10,000 to 100,000; 1 person (12.50%) was defrauded with an amount of more than 100,000.

**Fig. 10:**
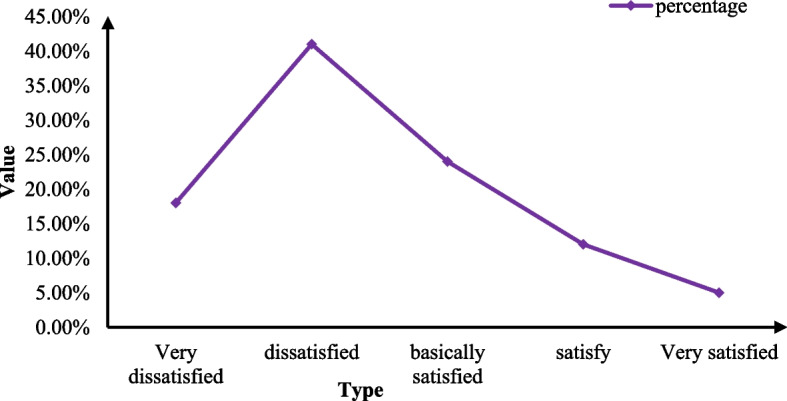
Citizens’ satisfaction survey on the government’s governance against internet fraud

In Fig. 10, 18 (18.00%) residents were very dissatisfied with the current situation, and 41 (41.00%) were dissatisfied with it; 24 people (24.00%) were basically satisfied with it, and 12 people (12.00%) were satisfied with it; 5 (5.00%) residents were very satisfied. A total of 59 residents were dissatisfied with the current situation, accounting for more than 59.00%.

In addition, it can be seen from Table 5 that 60 citizens showed a positive attitude towards the government’s anti-Internet fraud governance, while 40 citizens said they did not take positive actions. During the survey, some citizens also left comments in the governance suggestion column. The first is that publicity should be strengthened and multi-channel publicity should be carried out; the commonly used fraudulent methods should be made public, so that the public can have a perceptual understanding. The second is to accurately publicize high-risk areas such as communities and schools, and effectively protect vulnerable groups. The third is to simplify the reporting procedures and shorten the time for the public to report to the police.

**Table 5:** Whether the government has taken an active role in governance and combating cyber fraud

Problem	Option	Number of people	Percentage
Has the government taken an active role in combating cyber fraud?	Yes	60	60.00%
	no	40	40.00%
	total	100	100.00%

#### Comparison of 3 classes i.e. normal class, warning class and emergency class in the cyber fraud

**Fig. 11:**
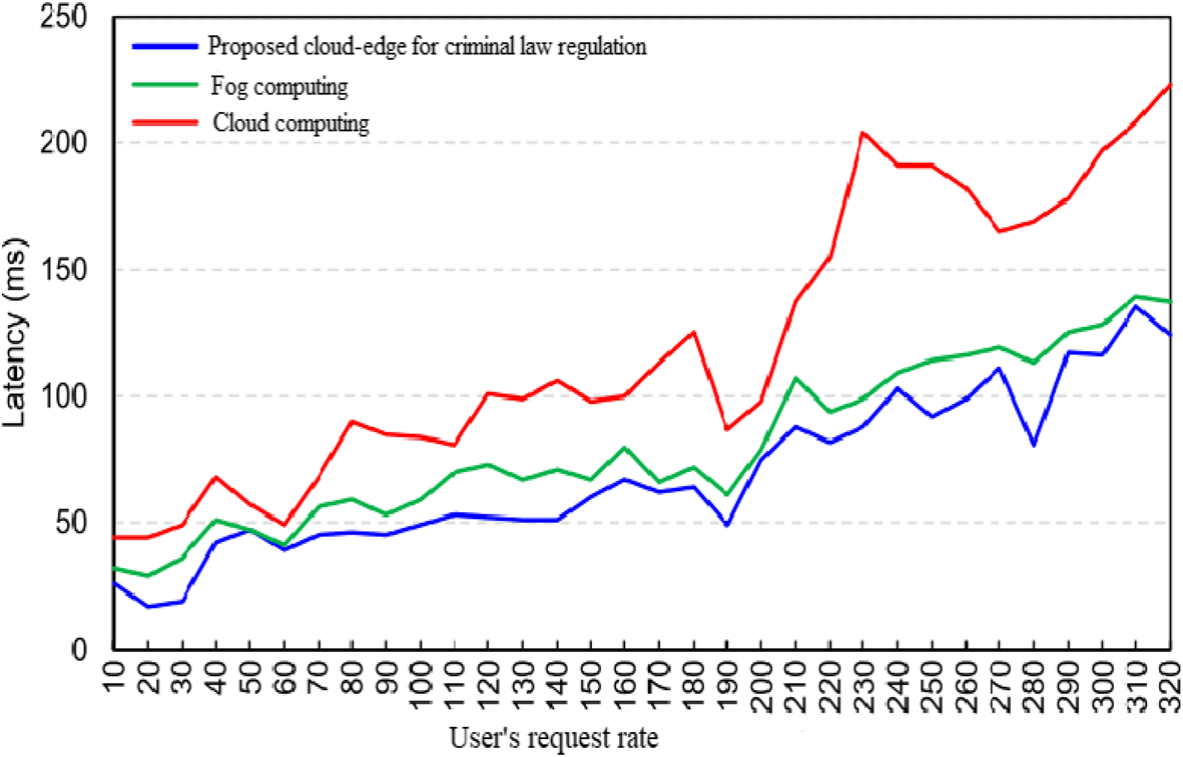
Average system utilization time per user request

**Fig. 12:**
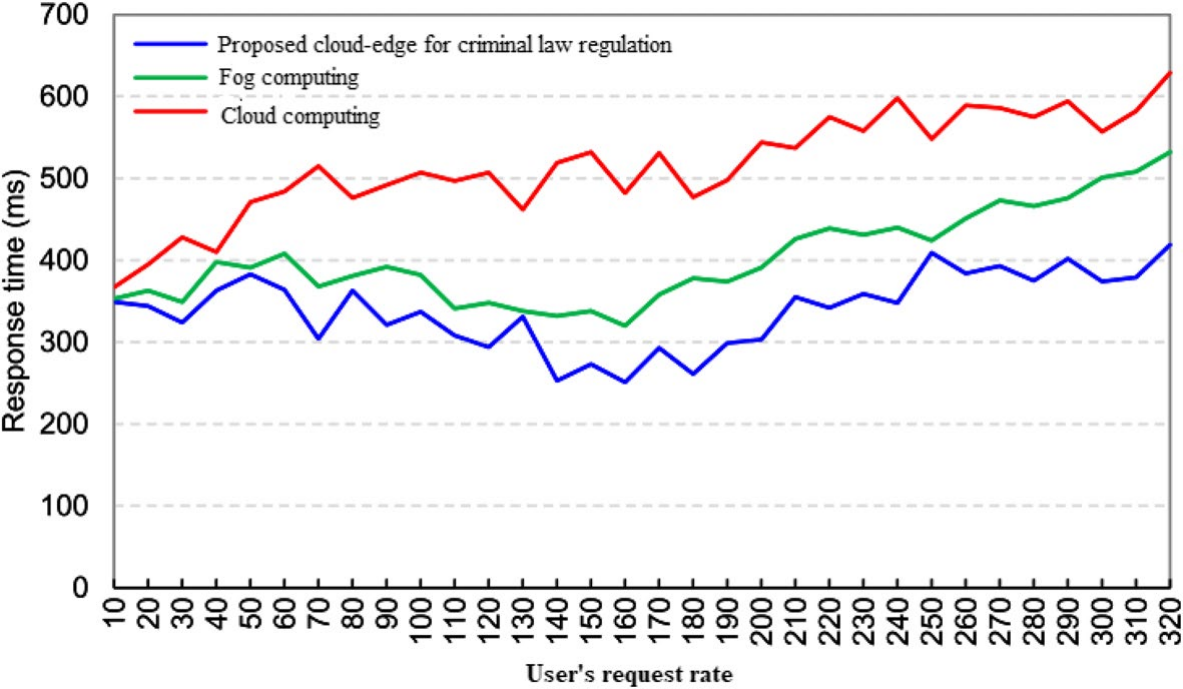
Average system latency per user request

This section compares the three normal classes warning, and emergency. Figure 11 shows a comparison of the average latency in the proposed model, fog computing, and cloud computing with different user request rates based on the above calculation. In comparison to cloud and fog computing, the prototype system reduced latency by 43.01% and 14.25%, respectively. Based on the above equations, Fig. 12 highlights a correlation between the average response time in the proposed system with fog and cloud computing. In comparison to cloud and fog computing, the proposed method decreased reaction time by 35.01% and 16.1%, respectively.

### Suggestions for low governance capacity

**Fig. 13:**
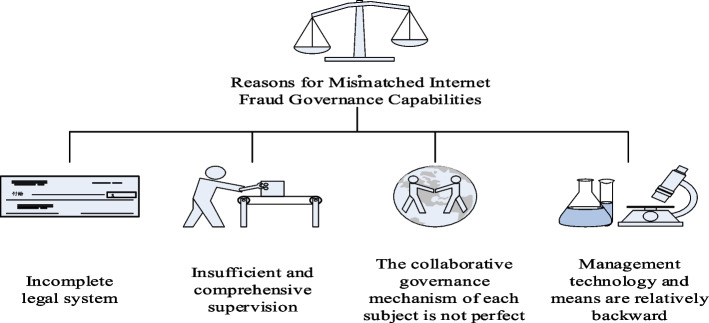
Reasons for a mismatch in cyber fraud governance capabilities

From the perspective of supervision, the volume of urban construction and network information platform supervision work in City Z has greatly increased. However, due to the continuous acceleration of urbanization in City Z, the work volume from various aspects such as vehicle production market, radio resources, communication lines, payment settlement market has greatly increased. Therefore, the supervision of cyber fraud governance in City Z still has the problem of incomplete and insufficient supervision. In addition, due to the cooperation of multiple departments such as capital flow, information flow mechanism, communication operators, commercial banks, and government departments, the multi-department cooperation mechanism in City Z has certain defects. If the outside world needs the cooperation of multiple departments, it would inevitably cause work confusion and reduce work efficiency [25]. Therefore, the reasons for the mismatch of network fraud governance capabilities are analyzed from the following perspectives in Fig. 13.

At present, cyber fraud crimes occur from time to time. However, China’s investigation and governance technology is relatively lagging behind, which makes China lack relevant technology and equipment in actual work, and causes China’s overall anti-fraud detection rate to be low. Internet fraud in China not only has technical problems in detection and governance, but also in prevention. In current China, due to deficiencies in prevention, detection, and governance technologies, it is more difficult to prevent and manage cyber fraud. The technical level of governance is also low, which hinders China’s efforts to combat cyber fraud.

**Fig. 14:**
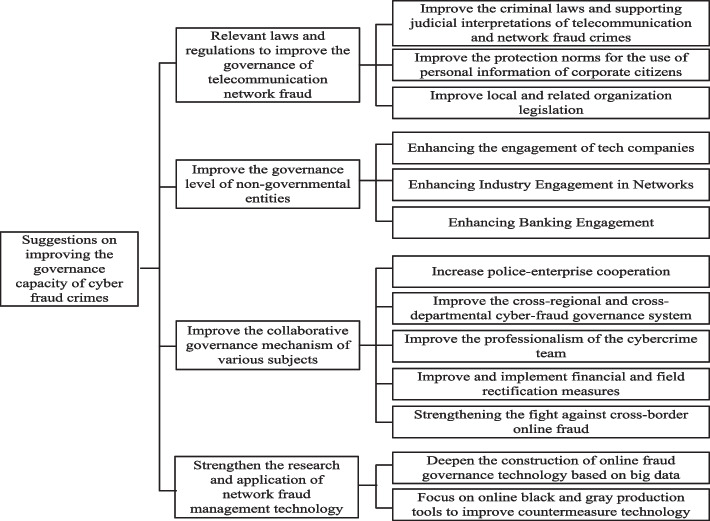
Suggestions for lifting

Some suggestions in Fig. 14 are given for the improvement of Internet fraud crime governance capabilities.

In the current era of big data information, the supervision of cyber fraud should be strengthened and effective historical tracking should be carried out. By grasping its basic characteristics and internal laws, its development trend can be predicted to a certain extent, thereby enhancing the prevention awareness of relevant departments. In view of the current new situation of cyber fraud, and according to the needs of combating crimes, it is necessary to establish and improve an integrated crime-fighting platform for public security organs and relevant departments, and attach importance to the further improvement of crime-fighting platforms.

### Improvement of criminal liability evaluation for internet fraud crimes

From a macro perspective, it is imperative to establish and improve the criminal liability assessment system for China’s Internet fraud crimes.

Whether it is traditional fraud or cyber fraud, public property and private property should be protected as an important criterion for measuring their criminal responsibility. Since traditional fraud models are single, small-scale frauds, the number of perpetrators can be used to judge whether the perpetrator is guilty or not. However, when using the Internet to conduct cyber fraud, the way of committing crimes presents the characteristics of one-to-many, multi-faceted, and multi-form, which is very different from the traditional form of fraud. Therefore, it cannot be measured only in terms of quantity, and the evaluation factors of criminal responsibility must be expanded.

It can start from three aspects: the expansion of the victim standard, the expansion of the object order standard, and the adjustment of the hierarchical relationship of the criminal responsibility evaluation factors. From the perspective of specific methods, it can start from the legislative and judicial aspects.

#### Legislative level

The amendments focus on the “amount” legislative model. At present, there are many problems in China’s assessment of responsibility for Internet fraud crimes, and the fundamental reason is that legislation is lagging behind. In today’s networked era, fraudsters can set up Trojan horses and mass fraudulent text messages on the Internet, and even use the Internet to make repeated calls. The amount of fraud can vary from a few thousand to millions or even tens of millions. These are incomparable to traditional fraud methods. Although the probability of each success is very low, once accumulated, it would bring huge harm to society, and may even violate property. Therefore, it is necessary to change the way of conviction based on the amount of money.

#### Judicial level

The construction of a criminal liability assessment system based on the number of people and objects. When the legislature uses “amount + circumstance” as a measure of criminal responsibility, judges should follow in the footsteps of the legislature. A more detailed judicial interpretation should be made to make it operational in judicial practice. The specific terms on the amount of money have already been stipulated in the relevant content. However, although there are legal interpretations in this regard, it is not very clear. Therefore, more detailed and scientific explanations are needed from judicial institutions to construct a criminal liability evaluation system of “person-time standard + object-time standard”.

## Conclusions

Internet fraud is a new type of fraud crime, which is different from traditional fraud. It is done through contactless means, online media, and new payment media. However, with the continuous updating of cyber fraud methods, the related forensic identification work is becoming more and more difficult. Through the study of a series of judicial cases, it is hoped that it would help people to understand related issues in judicial practice. At this stage, the process of cyber fraud crime governance is essentially a comprehensive systematic project, which requires the joint participation and cooperation of various parties. In the era of cloud-edge computing, cyber fraud is also changing with the times and the development of science and technology. In the face of the new trend of cyber fraud, it is necessary to effectively improve the governance mechanism of cyber fraud, carry out more targeted prevention and rectification work, and enhance the fight against cyber fraud. As a black product of the development of social information, the governance of cyber fraud needs to integrate the strength of the Chinese government and social organizations, constantly improve the governance system of cyber fraud, form a scientific and effective long-term governance mechanism, and safeguard the public’s interests at a higher level, to build a modern society of integrity and harmony.

## Data Availability

The data that support the findings of this study are available from the corresponding author upon reasonable request.
